# Spatio-temporal graph convolutional networks with transfer learning for continuous ground reaction force estimation in hemiparetic gait

**DOI:** 10.3389/fbioe.2026.1871692

**Published:** 2026-07-15

**Authors:** Qinghua Meng, Zhiyuan Yang, Yijia Xue, Luxing Zhou, Nan Zhang, Miaomiao Xiao, Xuequan Feng, Chunyu Bao

**Affiliations:** 1 Tianjin University of Sport, Tianjin, China; 2 The Neurosurgical Department of Tianjin First Central Hospital, Tianjin, China

**Keywords:** digital biomarker, gait analysis, ground reaction force, hemiparetic gait, rehabilitation engineering, spatio-temporal graph convolutional network, transfer learning

## Abstract

Three-dimensional ground reaction force (GRF) is an important biomechanical indicator of weight-bearing, propulsion, and bilateral asymmetry in hemiplegic gait. However, conventional GRF measurement relies on laboratory-based force plates, limiting its use in continuous rehabilitation assessment. A specific methodological challenge is to estimate continuous three-dimensional GRF in post-stroke hemiplegic gait without using force-plate signals as model input, while still preserving whole-body kinematic coordination and affected–unaffected side asymmetry. This study proposed a force-plate-independent, marker-based method for estimating continuous stance-phase three-dimensional GRF in patients with hemiplegia by combining a spatio-temporal graph convolutional network (ST-GCN) with two-stage transfer learning. Data were collected from 30 chronic stroke patients with hemiplegia and 60 healthy controls. The model used 39 raw Plug-in Gait markers as graph nodes, with 10-dimensional node features consisting of three-dimensional position, velocity, acceleration, and laterality encoding. The model was pretrained using healthy participant data and then fine-tuned and evaluated on hemiplegic gait data using leave-one-subject-out cross-validation. The main contribution of this work is the integration of marker-level body topology, explicit kinematic derivatives, pathological laterality encoding, and healthy-to-hemiplegic transfer learning within a unified ST-GCN framework. The proposed ST-GCN achieved Pearson’s r values of 0.984, 0.956, and 0.912, and rRMSE values of 5.24%, 8.15%, and 11.05% for vertical, anterior–posterior, and medio–lateral GRF prediction, respectively, outperforming MLP, 2D-CNN, BiLSTM, and lightweight Transformer baselines. Bland–Altman analysis further showed small mean biases for the first vertical peak force, anterior–posterior peak propulsive force, weight-bearing asymmetry index, and propulsion asymmetry index, with all participants falling within the 95% limits of agreement. These findings suggest that the proposed framework can reconstruct overall GRF waveform morphology and preserve group-level kinetic asymmetry features in hemiplegic gait. The method may provide a force-plate-independent, marker-based laboratory framework for kinetic gait assessment, but it should not yet be interpreted as a fully wearable or home-based clinical monitoring system.

## 1 Introduction

Post-stroke hemiplegia commonly leads to impaired lower-limb motor control, reduced gait efficiency, and asymmetries in bilateral weight-bearing and propulsive capacity (Kim and Eng, 2004; 2003). Three-dimensional ground reaction force (GRF) reflects weight-bearing, braking, propulsion, and lateral stability during the stance phase, and is therefore an important biomechanical indicator for evaluating hemiplegic gait and quantifying rehabilitation outcomes (Bowden et al., 2006; Balasubramanian et al., 2007; Kuwabara et al., 2025). In particular, the first vertical loading peak and the anterior–posterior propulsive peak are commonly used to assess insufficient affected-side weight bearing, impaired propulsion, and bilateral kinetic asymmetry (Kim and Eng, 2003; Bowden et al., 2006; Biere et al., 2025).

However, accurate GRF measurement typically relies on laboratory-based force plates, which are costly, spatially constrained, and difficult to use for continuous monitoring in community-based or home-based rehabilitation settings (Oh et al., 2013; Martínez-Pascual et al., 2023). Traditional inverse dynamics and musculoskeletal models can estimate GRF from kinematic data, but their outputs depend on joint center estimation, model scaling, segmental inertial parameters, and inverse kinematics calculations (Delp et al., 2007; Oh et al., 2013). In patients with hemiplegia, pathological characteristics such as spasticity, reduced muscle strength, pelvic compensation, and abnormal foot–ground contact may introduce substantial errors into standard human body models, thereby limiting their applicability to pathological gait analysis (Kim and Eng, 2004; Bowden et al., 2006).

Therefore, the specific problem addressed in this study is how to estimate continuous three-dimensional stance-phase GRF in patients with post-stroke hemiplegia from marker-level kinematic data, while preserving temporal dynamics, human body topology, and affected–unaffected side asymmetry. This problem is clinically relevant because hemiplegic gait is characterized by impaired weight bearing, reduced propulsion, and bilateral kinetic asymmetry, but direct force-plate measurement is difficult to apply repeatedly in routine rehabilitation assessment.

To overcome the spatial constraints of force plates and optical laboratories, several research groups have developed wearable sensing approaches for GRF or propulsion estimation. These approaches have used pressure-sensing insoles, instrumented shoes, load cells, and inertial measurement units (IMUs) to estimate vertical or three-dimensional GRF during walking (Martínez-Pascual et al., 2023; Kim et al., 2023; 2025), and recent studies have extended such systems to post-stroke propulsion estimation across walking speeds (Swaminathan et al., 2025) and real-time anterior–posterior GRF estimation using wearable sensors and deep learning (Nurse et al., 2025). Inertial motion capture alone has also been investigated for estimating GRF and moments during gait (Karatsidis et al., 2017). Compared with marker-based optical motion capture, wearable systems offer greater portability and a clearer pathway toward continuous community or clinical monitoring; however, they may be affected by sensor placement, calibration requirements, drift, footwear or insole fit, and reduced access to whole-body kinematic coordination, particularly for pathological gait and shear-force components. Conversely, marker-based motion capture provides high-resolution whole-body kinematic information that is useful for modeling multi-segment coordination and pathological asymmetry, but it remains laboratory dependent and less accessible in routine clinical settings.

In recent years, data-driven methods have provided new opportunities for learning the nonlinear mapping from kinematics to kinetics (Oh et al., 2013; Martínez-Pascual et al., 2023; Khan et al., 2024). Models such as MLPs (Rumelhart et al., 1986), CNNs (LeCun et al., 1998), LSTMs (Hochreiter and Schmidhuber, 1997), and Transformers (Vaswani et al., 2017) have been applied to GRF prediction tasks (Feng et al., 2025; Kim et al., 2023; 2025). However, these methods typically flatten human marker coordinates or transform them into regular grid-like representations, which may weaken the intrinsic spatial topology of the human kinematic chain (Feng et al., 2025). Therefore, within the broader landscape of wearable, markerless, and marker-based GRF estimation, an important methodological question is how to exploit the rich spatial information available from marker trajectories while reducing dependence on force plates. In hemiplegic gait, pronounced kinetic asymmetry and compensatory coordination exist between the affected and unaffected sides (Bowden et al., 2006; Balasubramanian et al., 2007). Therefore, an effective model should simultaneously characterize temporal dynamics, human body topology, and pathological laterality differences.

Spatio-temporal graph convolutional networks (ST-GCNs) can represent human markers as graph nodes and use graph edges to describe spatial relationships within anatomical structures or kinematic chains, thereby enabling joint feature extraction along both the temporal and spatial-topological dimensions (Yan et al., 2018; 2024). This property makes ST-GCNs well suited for continuous GRF estimation in hemiplegic gait, and graph-based models have already shown strong performance in pathological gait classification and lower-limb motion recognition (Zhao et al., 2024). In addition, the acquisition of hemiplegic gait data is challenging, sample sizes are often limited, and inter-individual variability is substantial. Training deep models directly on small pathological datasets can easily lead to overfitting. By contrast, data from healthy participants are relatively easier to obtain and contain fundamental kinematic–kinetic mapping relationships in typical walking. Such data can therefore serve as a pretraining source for transfer learning to alleviate the problem of insufficient pathological samples (Pan and Yang, 2010).

Therefore, the novelty of the present study lies in addressing a specific methodological gap in hemiplegic GRF estimation: how to preserve whole-body marker topology, pathological laterality information, and temporal dynamics within a unified force-plate-independent, marker-based framework. Unlike conventional models that flatten marker trajectories or wearable approaches that rely primarily on local sensor signals, the proposed ST-GCN directly represents 39 Plug-in Gait markers as graph nodes and learns spatio-temporal relationships among anatomical marker trajectories. This design allows the model to estimate continuous three-dimensional GRF waveforms while explicitly considering affected-side and unaffected-side kinetic asymmetry in post-stroke hemiplegic gait.

Based on these considerations, this study proposes a continuous three-dimensional GRF estimation framework that integrates ST-GCN with two-stage transfer learning. The proposed method uses 39 raw Plug-in Gait markers as graph nodes and directly establishes an end-to-end mapping from marker-level kinematics to continuous GRF waveforms. The model input consists of three-dimensional marker position, velocity, acceleration, and laterality encoding (p) , forming a 10-dimensional node feature representation to enhance the model’s sensitivity to kinetic changes and left–right asymmetry in hemiplegic gait. During training, the model is first pretrained using data from 60 healthy participants and then fine-tuned and tested using leave-one-subject-out cross-validation (LOSO-CV) on data from 30 patients with hemiplegia. The proposed model is further compared with baseline models, including MLP, 2D-CNN, BiLSTM, and a lightweight Transformer.

The scope of this study is limited to offline, laboratory-based estimation of stance-phase three-dimensional GRF during level walking using 39 Plug-in Gait markers as input. The present work does not aim to provide a fully wearable or home-based clinical monitoring system; rather, it evaluates a force-plate-independent, marker-based laboratory framework and its ability to reconstruct GRF waveforms and clinically relevant kinetic asymmetry features in patients with hemiplegia.

In this way, the present work contributes to the literature by extending GRF estimation from general gait or wearable-sensor settings toward topology-aware, marker-based kinetic modeling of post-stroke hemiplegic gait.

The main contributions of this study to the literature are as follows:

1. This study develops a marker-level ST-GCN model that uses 39 raw Plug-in Gait markers as graph nodes, thereby preserving human body topology and avoiding the loss of spatial relationships caused by directly flattening marker trajectories.
2. This study introduces a 10-dimensional node feature representation that combines position, velocity, acceleration, and laterality encoding (p) , allowing the model to explicitly distinguish affected-side, unaffected-side, and midline nodes and to represent asymmetric kinetic characteristics in hemiplegic gait.
3. This study adopts a two-stage transfer learning strategy that combines pretraining on healthy participants with LOSO-CV-based fine-tuning on patients with hemiplegia, and systematically compares the proposed model with multiple baseline models to evaluate generalization performance in unseen patients.

## 2 Methods

### 2.1 Participant characteristics and experimental protocol

A total of 90 participants were enrolled in this study, including 30 patients with post-stroke hemiplegia (hemiplegia group) and 60 healthy adults without gait disorders (healthy group). The inclusion criteria for the hemiplegia group were as follows: (1) unilateral stroke onset more than 6 months before enrollment, corresponding to the chronic stage; (2) the ability to walk independently for more than 10 m, with assistive devices permitted but without physical assistance from another person, and a Functional Ambulation Category score greater than 3; and (3) no severe cognitive impairment or concomitant lower-limb osteoarticular disorders. Participants in the healthy group were required to have no history of neuromuscular disease.

All participants provided written informed consent before testing, and the study protocol was approved by the Ethics Review Committee. The core baseline clinical characteristics of the participants are summarized in Table 1. The natural epidemiological differences in age and walking speed between the healthy and hemiplegia groups were addressed in the subsequent data augmentation and transfer learning strategies.

**TABLE 1:** Baseline clinical and demographic characteristics of the participants.

Characteristics	Hemiplegia group	Healthy group
	(n=30)	(n=60)
Age (years)	58.4±9.2	26.5±4.1
Sex (M/F)	19/11	28/32
Body mass index (BMI, kg/ $m^{2}$ )	24.6±3.1	23.1±2.8
Paretic side (L/R)	16/14	N/A
Self-selected walking speed (m/s)	0.45±0.18	1.25±0.12
FAC score (3/4/5)	8/15/7	N/A

Differences in age and self-selected walking speed were observed between the healthy and hemiplegia groups, reflecting the natural demographic and gait-related differences between young healthy participants and patients with chronic hemiplegia. In this study, data from the healthy group were used only for pretraining to learn the fundamental kinematic–kinetic mapping relationships in typical walking. The model was subsequently fine-tuned using data from patients with hemiplegia to adapt it to pathological gait characteristics, including insufficient weight-bearing, reduced propulsion, and bilateral asymmetry.

Gait data were collected in two independent biomechanics laboratories, which were used to acquire data from the 60 healthy participants and the 30 patients with hemiplegia, respectively. To minimize potential systematic errors introduced by cross-laboratory differences, both collection centers used the same hardware configuration and standardized testing protocol. Whole-body kinematic data were collected using a 10-camera infrared optical motion capture system (Qualisys, Gothenburg, Sweden) at a sampling frequency of 100 Hz. During testing, all participants wore tight-fitting clothing to reduce soft tissue artifacts, and 39 reflective markers were attached to anatomical landmarks strictly following the full-body Plug-in Gait (PiG) protocol. Synchronized kinetic data, namely, three-dimensional GRF, were recorded at 1000 Hz using two independent three-dimensional force plates (Kistler, Winterthur, Switzerland) embedded flush with the central region of the level walkway.

All participants were instructed to walk naturally along the walkway at their self-selected comfortable speed. After repeated trials, at least five valid complete gait cycles were retained for each participant.

### 2.2 Data preprocessing and multi-gradient physics-based augmentation

To eliminate sampling-rate differences across the multimodal acquisition system and to strictly align kinematic features with kinetic outputs in the temporal domain, a unified data preprocessing pipeline was established in this study. First, the three-dimensional coordinate data of the 39 raw markers were smoothed using a fourth-order zero-phase Butterworth low-pass filter with a cutoff frequency of 15 Hz, to reduce the influence of skin-related soft tissue artifacts and high-frequency measurement noise on the calculation of velocity and acceleration. The synchronously collected three-dimensional ground reaction force (GRF) signals were also processed using a fourth-order zero-phase Butterworth low-pass filter, with a cutoff frequency of 25 Hz, and were then downsampled to 100 Hz to match the kinematic data.

The filter parameters were selected with reference to standard gait biomechanics processing procedures and were further determined based on the spectral characteristics of the present dataset and preliminary experimental results. This setting was intended to suppress high-frequency noise while preserving the main waveform characteristics of stance-phase GRF. Subsequently, in the real absolute time domain, the instantaneous first-order derivatives, namely, velocity components $\left(v_{x},v_{y},v_{z}\right)$ , and second-order derivatives, namely, acceleration components $\left(a_{x},a_{y},a_{z}\right)$ , of each marker were calculated using finite differences, thereby explicitly preserving the most direct Newtonian kinetic information. All GRF data were normalized to body weight and expressed as %BW. The stance phase was segmented from heel strike to toe-off and uniformly resampled to 101 frames, representing 0%–100% of the stance phase.

To mitigate the risk of overfitting caused by the limited sample size and cadence variability in patients with hemiplegia, multi-gradient temporal data augmentation was applied only to the training set. Specifically, time scaling factors of −2.5% , +2.5% , −7.5% , and +7.5% were applied to the stance-phase sequences to simulate mild to moderate fluctuations in cadence and stance-phase duration during natural walking. In preliminary experiments, these augmentation magnitudes did not introduce apparent distortion of pathological waveforms. This augmentation increased the temporal diversity of the training samples while preserving key characteristics of hemiplegic gait, including insufficient weight-bearing, reduced propulsion, and bilateral asymmetry. No temporal deformation was applied to the validation or test sets, to avoid data leakage and ensure the objectivity of model generalization evaluation. Thus, each original training sample was expanded into five samples, consisting of one original sample and four time-scaled samples.

Finally, cubic spline interpolation was used to uniformly resample all temporally deformed training sequences and the unchanged test sequences to fixed-length input matrices of 101 frames, representing 0%–100% of the stance phase. This procedure completed the standardized preprocessing pipeline for graph network input data.

### 2.3 Graph topology construction and 10-dimensional pathological node encoding

**FIGURE 1:**
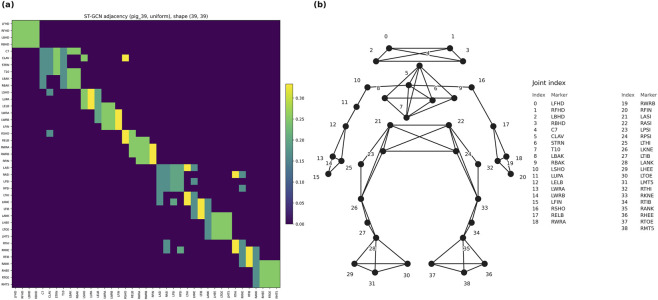
Definition of graph neural network nodes. **(a)** Initialization of the spatial adjacency matrix. **(b)** Schematic illustration of the marker-node topology and anatomical connectivity.

Instead of using a conventional inverse kinematics (IK) model, which may introduce cumulative errors, this study directly defined the 39 raw reflective markers as graph neural network nodes, or vertices (V=39) . Because skin-mounted markers do not have absolute rigid hinge-like physical connections comparable to skeletal joints, the spatial adjacency matrix (A) was initialized according to human anatomical segments, such as marker clusters on the thigh, shank, and trunk. This matrix was used to represent the initial physical connectivity for feature propagation, as shown in Figure 1.

To enable the network to explicitly perceive left–right asymmetry and compensatory mechanisms in hemiplegic gait, a pathological feature encoding with laterality information, denoted as p , was designed at the input level. For any node i among the 39 markers, $p_{i}$ was assigned a value of 1 for the affected side, −1 for the unaffected side, and 0 for midline nodes. For healthy participants, all nodes were assigned $p_{i}=0$ . Consequently, the input feature vector of a single node at any time step was expanded to 10 dimensions (C=10) , expressed as:

$$x_{i}={\left(x,y,z,v_{x},v_{y},v_{z},a_{x},a_{y},a_{z},p\right)}_{i}.$$ (1)

### 2.4 Spatio-temporal graph convolutional network

**FIGURE 2:**
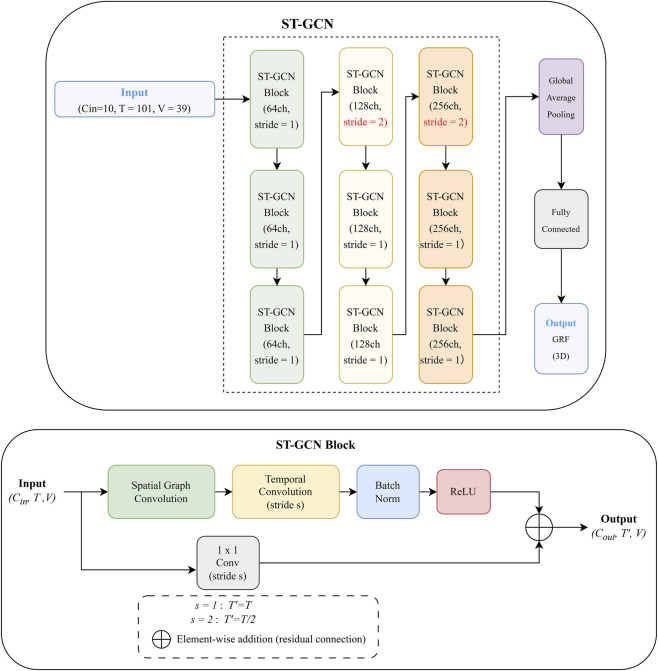
Architecture of the ST-GCN model.

To model the complex nonlinear relationship between multi-marker kinematic features during the stance phase and three-dimensional ground reaction force (GRF), this study employed a spatio-temporal graph convolutional network (ST-GCN) as the prediction model, as shown in Figure 2. The network represents each gait sample as a temporal graph signal defined on the human body graph topology. It learns coordinated relationships among different markers in the spatial dimension and extracts dynamic changes during the stance phase in the temporal dimension, thereby enabling end-to-end regression of continuous GRF waveforms.

Let the input feature be denoted as $X\in R^{C\times T\times V}$ , where C , T , and V represent the number of feature channels, temporal frames, and graph nodes, respectively. The network output is denoted as $\hat{Y}\in R^{3\times T}$ , corresponding to the GRF components in three directions.

#### 2.4.1 Network architecture

The ST-GCN adopted in this study was constructed by stacking multiple spatio-temporal graph convolutional units. Each unit consisted of a spatial graph convolution, a temporal convolution, and a residual connection. For the input feature of the l -th layer, denoted as $H^{\left(l\right)}$ , the output can be expressed as:

$$H^{\left(l+1\right)}=\sigma \left(T^{\left(l\right)}\left(G^{\left(l\right)}\left(H^{\left(l\right)}\right)\right)+R^{\left(l\right)}\left(H^{\left(l\right)}\right)\right),$$ (2)

where $G^{\left(l\right)}\left(\cdot \right)$ and $T^{\left(l\right)}\left(\cdot \right)$ denote the spatial graph convolution and temporal convolution at the l -th layer, respectively; $R^{\left(l\right)}\left(\cdot \right)$ denotes the residual mapping; and σ(⋅) represents the ReLU activation function.

#### 2.4.2 Spatial graph convolution

Spatial graph convolution was used to aggregate neighborhood features of graph nodes at each time step, thereby modeling the spatial dependencies among markers. Let the adjacency matrix be denoted as $A\in R^{V\times V}$ . Because only a single adjacency matrix was used in this study, K=1 was adopted for the spatial convolution. To improve numerical stability, self-connections were first added to the adjacency matrix, followed by normalization:

$$\tilde{A}=A+I,\;{\tilde{D}}_{ii}=\underset{j}{\sum }{\tilde{A}}_{ij},\;\hat{A}={\tilde{D}}^{-\frac{1}{2}}\tilde{A}{\tilde{D}}^{-\frac{1}{2}}.$$ (3)

The spatial graph convolution at the l -th layer can then be expressed as:

$$Z^{\left(l\right)}=W_{g}^{\left(l\right)}H^{\left(l\right)}\hat{A},$$ (4)

where $W_{g}^{\left(l\right)}$ is the learnable weight matrix and $Z^{\left(l\right)}$ is the output of the spatial convolution. In node-wise form, this operation can be written as:

$$z_{i,t}^{\left(l\right)}=\overset{V}{\underset{j=1}{\sum }}{\hat{A}}_{ij}W_{g}^{\left(l\right)}h_{j,t}^{\left(l\right)}.$$ (5)

This indicates that the output of node i at time step t is obtained by weighted aggregation of the features of its neighboring nodes. This operation enables the network to extract spatial coordination patterns among different markers under the constraints of the anatomical graph topology.

#### 2.4.3 Temporal convolution

After spatial feature aggregation, the network further performs convolution along the temporal dimension to extract dynamic evolution patterns within the stance phase. Given the output of the spatial graph convolution, denoted as $Z^{\left(l\right)}$ , the temporal convolution can be expressed as:

$$U^{\left(l\right)}={Conv}_{\left(K_{t},1\right)}\left(Z^{\left(l\right)}\right),$$ (6)

where $K_{t}$ denotes the temporal convolution kernel size. The convolution is applied only along the temporal dimension and does not alter the node dimension.

For any node i and output time step τ , the temporal convolution can be written as:

$$u_{i,\tau }^{\left(l\right)}=\overset{K_{t}-1}{\underset{m=0}{\sum }}W_{t}^{\left(l\right)}\left(m\right)z_{i,\tau +m-\left\lfloor K_{t}/2\right\rfloor }^{\left(l\right)}+b^{\left(l\right)},$$ (7)

where $W_{t}^{\left(l\right)}$ represents the learnable parameters of the temporal convolution kernel, and $b^{\left(l\right)}$ is the bias term. This operation is used to capture local temporal motion patterns within the stance phase. If the stride is denoted as s , the output temporal length is given by:

$$T^{\prime }=\left\lfloor \frac{T+2P-K_{t}}{s}\right\rfloor +1,$$ (8)

where P is the padding size. When s=2 , temporal downsampling can be achieved, thereby enlarging the temporal receptive field and reducing computational complexity.

#### 2.4.4 Residual connection and output

To improve the training stability of the deep network, a residual connection was introduced into each ST-GCN unit, defined as:

$$R^{\left(l\right)}\left(H^{\left(l\right)}\right)=\left\{\begin{array}{cc} H^{\left(l\right)}, & \text{if }C_{l}=C_{l+1}\text{ and }s=1,\\ {Conv}_{1\times 1}\left(H^{\left(l\right)}\right), & \text{otherwise}. \end{array}\right.$$ (9)

When the input and output dimensions were consistent, an identity mapping was used. Otherwise, a 1×1 convolution was applied to match the channel dimension or temporal resolution. Finally, after global pooling and linear projection of the high-level network features, the predicted output was obtained as:

$$\hat{Y}=f_{\theta }\left(X,\hat{A}\right),$$ (10)

where $f_{\theta }\left(\cdot \right)$ denotes the complete ST-GCN model.

Overall, the network extracts topological coordination relationships among markers through spatial graph convolution and captures continuous dynamic evolution features within the stance phase through temporal convolution, thereby enabling effective prediction of three-dimensional GRF waveforms.

### 2.5 Evaluation metrics

To evaluate the generalization performance of the model in unseen patients with hemiplegia, leave-one-subject-out cross-validation (LOSO-CV) was performed on data from 30 patients with hemiplegia. Continuous GRF prediction performance was assessed using Pearson’s correlation coefficient (Pearson’s r ) and relative root mean square error (rRMSE). Pearson’s r was used to evaluate the waveform similarity between the predicted and measured GRF curves, whereas rRMSE was used to quantify the prediction error in amplitude. All metrics were calculated separately for the vertical, anterior–posterior, and medio–lateral GRF components and then averaged at the participant level.

The rRMSE was defined as follows:

$$rRMSE\left(\%\right)=\frac{\sqrt{\frac{1}{T}\sum_{t=1}^{T}{\left(Y_{t}-{\hat{Y}}_{t}\right)}^{2}}}{Y_{\max}-Y_{\min}}\times 100\%,$$ (11)

where $Y_{t}$ and ${\hat{Y}}_{t}$ denote the measured and predicted GRF at the t -th frame, respectively; T is the number of temporal frames in the stance phase; and $Y_{\max}-Y_{\min}$ represents the amplitude range of the measured GRF curve.

To further evaluate the model’s ability to predict clinically relevant discrete kinetic features, the first vertical peak force $\left({vGRF}_{\max}\right)$ and the anterior–posterior propulsive peak force $\left({apGRF}_{\text{prop}}\right)$ were extracted from the continuous GRF curves. Based on these features, the asymmetry index (ASI) between the affected and unaffected sides was calculated as follows:

$$ASI\left(\%\right)=\frac{\left|V_{\text{paretic}}-V_{\text{non}-\text{paretic}}\right|}{0.5\times \left(V_{\text{paretic}}+V_{\text{non}-\text{paretic}}\right)}\times 100\%,$$ (12)

where V represents the selected GRF peak parameter, and $V_{\text{paretic}}$ and $V_{\text{non}-\text{paretic}}$ represent the corresponding parameters on the affected and unaffected sides, respectively. Finally, Bland–Altman analysis was used to compare the agreement between model-predicted features and force-plate-measured features, and the mean bias and 95% limits of agreement were reported. The node feature definition, ST-GCN operations, temporal and residual mappings, model output, rRMSE, and ASI calculations are formally defined in Equations 1–12.

### 2.6 Experimental setup and network training details

All deep learning models in this study, including the proposed ST-GCN and all baseline models, were implemented using the PyTorch framework. Training and inference were performed on a workstation equipped with a single NVIDIA GeForce RTX 3090 GPU with 24 GB of VRAM. To ensure the comparability of model evaluation, all models used the same data preprocessing pipeline, training augmentation strategy, and two-stage transfer learning procedure.

In the first-stage pretraining, data from the 60 healthy participants were divided into training and validation sets at the participant level with a ratio of 8:2. In this stage, the model was trained from scratch to learn the fundamental mapping relationship between kinematic features and GRF waveforms in typical walking, thereby providing initial weights for subsequent fine-tuning on hemiplegic gait data. The maximum number of pretraining epochs was set to 200, with a batch size of 64. The Adam optimizer was used with an initial learning rate of $1\times 10^{-3}$ , together with a cosine annealing strategy for learning rate adjustment. After pretraining, the model parameters that achieved the best validation performance were saved.

In the second-stage fine-tuning, leave-one-subject-out cross-validation (LOSO-CV) was performed on data from the 30 patients with hemiplegia to evaluate the model’s generalization ability to unseen patients. In each fold, one patient with hemiplegia was completely held out as the test subject, while the remaining 29 patients were used for model fine-tuning and validation. Specifically, among the 29 non-test patients, 26 patients were used for fine-tuning training, and three patients were used as the validation set for early stopping and model selection. All gait cycles from the test patient were excluded from training, validation, data augmentation, and model selection to avoid subject-level data leakage.

During fine-tuning, the model was initialized with the weights pretrained on healthy gait data, and a differential learning rate strategy was adopted. Specifically, the learning rate of the spatio-temporal feature extraction backbone was set to $1\times 10^{-5}$ to preserve the general spatio-temporal representations learned during pretraining. The learning rate of the 1D-CNN regression head was set to $1\times 10^{-3}$ to enhance the model’s adaptability to hemiplegia-specific kinetic patterns. The maximum number of epochs in each fold was set to 100, with weight decay of $1\times 10^{-4}$ and an early stopping mechanism. If the validation mean squared error (MSE) did not decrease for 20 consecutive epochs, training was terminated early, and the model weights with the best validation performance in that fold were saved for testing.

In addition, to reduce the influence of differences in model capacity on the comparison results, the network sizes of the baseline models, including MLP, 2D-CNN, BiLSTM, and a lightweight Transformer, were controlled within a parameter range similar to that of the ST-GCN, approximately 2.5M–3.0M parameters. All baseline models followed the same training, validation, and test partitions, optimizer settings, data augmentation strategy, and two-stage transfer learning procedure as the ST-GCN, thereby improving the comparability of model performance evaluation.

## 3 Results

### 3.1 Overall prediction performance evaluation

To evaluate the performance of the proposed framework, the ST-GCN was systematically compared with four mainstream baseline models, namely, MLP, 2D-CNN, BiLSTM, and a lightweight Transformer, for predicting continuous three-dimensional GRF trajectories under the leave-one-subject-out cross-validation (LOSO-CV) framework. To ensure a fair comparison, the total number of parameters in all models was strictly controlled within the same order of magnitude, approximately 2.5M–3.0M, and all models adopted the differential learning rate-based transfer fine-tuning strategy described above. The overall model comparison results are summarized in Table 2.

**TABLE 2:** Quantitative evaluation of three-dimensional GRF prediction performance of different deep learning models under LOSO-CV.

Force component	Model	Pearson’s r	rRMSE (%)
Vertical $\left(F_{z}\right)$	MLP	0.892±0.045	11.84±3.21
	2D-CNN	0.931±0.032	8.65±2.34
	Transformer	0.945±0.028	7.32±2.11
	BiLSTM	0.968±0.021	6.45±1.82
	ST-GCN (Ours)	0.984±0.012	5.24±1.15
Anterior–Posterior $\left(F_{y}\right)$	MLP	0.821±0.062	16.27±3.88
	2D-CNN	0.874±0.048	13.08±2.95
	Transformer	0.892±0.041	11.95±2.64
	BiLSTM	0.925±0.035	10.12±2.15
	ST-GCN (Ours)	0.956±0.022	8.15±1.54
Medio–Lateral $\left(F_{x}\right)$	MLP	0.735±0.104	20.15±4.76
	2D-CNN	0.788±0.082	16.72±3.65
	Transformer	0.814±0.065	14.88±3.12
	BiLSTM	0.867±0.052	13.04±2.87
	ST-GCN (Ours)	0.912±0.038	11.05±2.18

Bold values indicate the best-performing model for each force component and metric.

A critical indicator of the biomechanical utility of a model is its ability to predict shear force components, including the anterior–posterior component related to braking and propulsion and the medio–lateral component associated with lateral balance. In these two challenging directions, the performance of MLP and 2D-CNN decreased markedly. For example, the medio–lateral rRMSE of MLP reached 20.15% . This finding suggests that flattened inputs or regular grid-like representations may be insufficient to preserve the non-Euclidean topological relationships among human body markers. By using topology-based spatial aggregation operators, the ST-GCN can implicitly model the physical kinetic chain from distal limb segments to the trunk, achieving a high correlation of r=0.956 in the anterior–posterior direction. Compared with 2D-CNN, the rRMSE in this direction was reduced by approximately 37%.

This study further included parameter-matched BiLSTM and lightweight Transformer models as strong temporal baseline models. Although Transformer models have shown strong performance in large-scale domains such as natural language processing, the lightweight Transformer did not exceed the conventional BiLSTM model in the present continuous GRF prediction task, with vertical rRMSE values of 7.32% and 6.45% , respectively. One possible explanation is that global self-attention mechanisms may require larger datasets to fully exploit model representational capacity. In contrast, the ST-GCN incorporates prior human connectivity as a graph-based structural constraint and uses 10-dimensional physics-informed node features. This design not only reduces the effective parameter search space but also enables the model to learn the underlying Newtonian kinematic–kinetic mapping with high data efficiency.

### 3.2 Comparison of ST-GCN prediction performance between the affected and unaffected sides

Given the pronounced bilateral kinetic asymmetry in hemiplegic gait, the performance of the ST-GCN model was further compared between affected-side and unaffected-side stance-phase three-dimensional GRF prediction. Gait cycles from each patient in the test set were categorized by side, and Pearson’s r and rRMSE were calculated at the participant level. Differences in prediction performance between the two sides were assessed using paired statistical tests, as shown in Table 3.

**TABLE 3:** Comparison of ST-GCN prediction performance for stance-phase three-dimensional GRF between the unaffected and affected sides.

Force component	Metric	Unaffected side	Affected side	p -value
Vertical $\left(F_{z}\right)$	Pearson’s r	0.989±0.008	0.978±0.015	$0.021^{*}$
	rRMSE (%)	4.65±1.12	5.83±1.65	$0.008^{**}$
Anterior–Posterior $\left(F_{y}\right)$	Pearson’s r	0.965±0.012	0.945±0.024	$0.015^{*}$
	rRMSE (%)	7.32±1.45	8.98±2.15	$<0.001^{***}$
Medio–Lateral $\left(F_{x}\right)$	Pearson’s r	0.925±0.018	0.898±0.035	$<0.001^{***}$
	rRMSE (%)	9.85±2.10	12.25±3.45	$<0.001^{***}$

Data are presented as participant-level mean ± standard deviation. Differences between the unaffected and affected sides were compared using paired statistical tests. ^*^ p<0.05 , ^**^ p<0.01 , and ^***^ p<0.001 .

Overall, the ST-GCN achieved high prediction agreement on both sides, although performance was lower on the affected side. For the vertical component $\left(F_{z}\right)$ , Pearson’s r was 0.989±0.008 on the unaffected side and 0.978±0.015 on the affected side, with rRMSE values of 4.65±1.12% and 5.83±1.65% , respectively. For the anterior–posterior component $\left(F_{y}\right)$ , the affected side also showed lower correlation and higher error, with rRMSE increasing from 7.32±1.45% on the unaffected side to 8.98±2.15% on the affected side. The greatest side-to-side difference was observed for the medio–lateral component $\left(F_{x}\right)$ , where the affected-side rRMSE reached 12.25±3.45% , compared with 9.85±2.10% on the unaffected side.

These results indicate that GRF prediction for the affected side is more challenging than for the unaffected side, particularly for the anterior–posterior propulsive and medio–lateral force components. This may be related to reduced muscle strength, unstable stance-phase control, insufficient propulsion, and greater inter-patient variability in compensatory strategies on the affected side. Nevertheless, the ST-GCN maintained high waveform agreement for affected-side three-dimensional GRF prediction, achieving Pearson’s r values of 0.978 and 0.945 for the vertical and anterior–posterior components, respectively. This suggests that the model can effectively capture the main kinetic characteristics related to affected-side weight-bearing and propulsion, providing a basis for subsequent extraction of bilateral asymmetry metrics from predicted GRF.

### 3.3 Continuous GRF trajectory analysis

**FIGURE 3:**
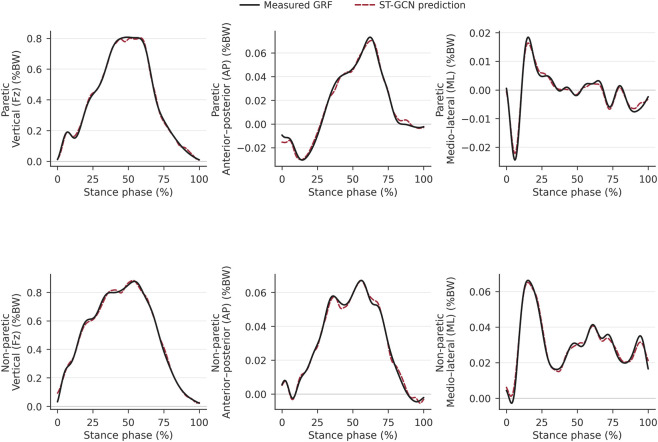
Comparison between measured and model-predicted continuous three-dimensional GRF trajectories on the affected and unaffected sides in a representative patient with hemiplegia.

To visually demonstrate the model’s ability to capture pathological characteristics of hemiplegic gait, Figure 3 shows the measured force-plate data and model-predicted trajectories during the stance phase of the affected and unaffected sides in a representative patient with hemiplegia.

As shown in Figure 3, the ST-GCN model closely fitted the abnormal waveform patterns of the affected-side GRF in the patient with hemiplegia. In particular, for the vertical GRF on the affected side, the model captured hemiplegic gait characteristics such as waveform flattening, reduced peak-to-valley differences, and the absence of the typical healthy double-peak pattern. These findings indicate that the model can reflect abnormal changes in the affected-side weight-bearing pattern during the stance phase. The high agreement with non-standard pathological waveforms suggests that the model did not simply memorize an average template derived from healthy participants, but instead learned individualized kinetic compensatory patterns.

### 3.4 Prediction performance for clinical discrete features and asymmetry metrics

**FIGURE 4:**
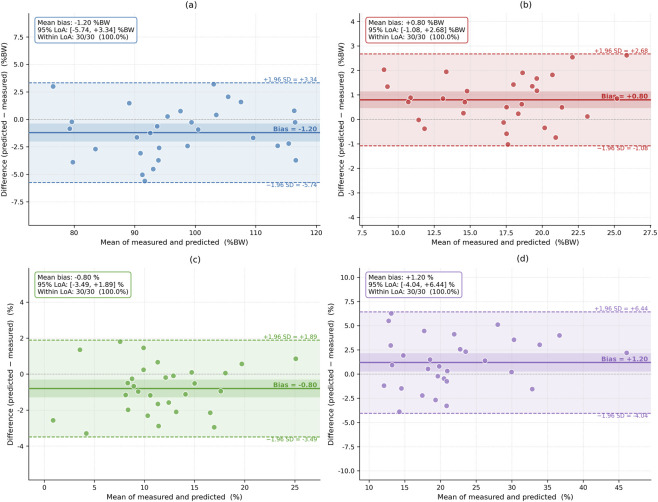
Bland–Altman agreement analysis for prediction of clinical discrete features and bilateral asymmetry metrics using the ST-GCN model. **(a)** First vertical peak_Fz1_. **(b)** Propulsion peak_Fy,max_. **(c)** Weight-bearing asymmetry_ASIF₂1_. **(d)** Propulsion asymmetry ASI_Fy,max_

To further evaluate the clinical utility of the model in gait analysis, discrete kinetic indicators commonly used in hemiplegic gait assessment were extracted from the predicted continuous GRF curves, including the first vertical peak force $\left(F_{z1}\right)$ and the anterior–posterior peak propulsive force $\left(F_{y,\max}\right)$ . Given the pronounced bilateral kinetic asymmetry in hemiplegic gait, these peak metrics were extracted separately from the affected- and unaffected-side stance phases. The weight-bearing asymmetry index $\left(ASI_{\mathrm{Fz1}}\right)$ and propulsion asymmetry index $\left(ASI_{Fy,\max}\right)$ were then calculated to quantify side-to-side differences in weight-bearing and propulsive capacity. All discrete features were averaged at the participant level and compared with force-plate measurements using Bland–Altman agreement analysis, as shown in Figure 4.

For the absolute peak metrics, the ST-GCN showed small systematic biases in predicting both $F_{z1}$ and $F_{y,\max}$ . The mean bias for $F_{z1}$ was −1.20% BW, with 95% limits of agreement (LoA) ranging from −5.74% to +3.34% BW. For $F_{y,\max}$ , the mean bias was +0.80% BW, with 95% LoA ranging from −1.08% to +2.68% BW. For both metrics, the predictions of all 30 participants fell within the 95% LoA, indicating that the model could stably estimate the vertical loading peak and anterior–posterior propulsive peak in hemiplegic gait.

For the bilateral asymmetry metrics, the ST-GCN also demonstrated good predictive agreement. The mean bias of $ASI_{\mathrm{Fz1}}$ was −0.80% , with 95% LoA ranging from −3.49% to +1.89% . The mean bias of $ASI_{Fy,\max}$ was +1.20% , with 95% LoA ranging from −4.04% to +6.44% . For both ASI metrics, all 30 participants again fell within the 95% LoA, suggesting that the model could not only estimate unilateral kinetic peaks but also preserve the bilateral asymmetry characteristics of weight-bearing and propulsion in patients with hemiplegia.

The scatter distributions showed no apparent increase in prediction error with increasing measurement magnitude, and no proportional bias was observed. Overall, the Bland–Altman analysis indicates that the ST-GCN model achieved agreement with force-plate measurements in quantifying key clinical discrete features and bilateral asymmetry metrics. The clinical interpretation of these error magnitudes was further contextualized in the Discussion with reference to published minimal detectable change (MDC) and smallest real difference (SRD) values for post-stroke GRF metrics.

### 3.5 Ablation study of core mechanisms

To evaluate the contribution of different modules to model performance, an ablation study was conducted under the leave-one-subject-out cross-validation framework. Two key designs were examined: the 10-dimensional node input features and the differential learning rate-based transfer fine-tuning strategy. Four model configurations were tested: Model A used only three-dimensional position coordinates (x,y,z) as input and was trained from scratch on data from patients with hemiplegia; Model B added velocity, acceleration, and laterality encoding to form 10-dimensional node features and was trained from scratch; Model C used 10-dimensional node features with pretraining on healthy participant data, followed by fine-tuning on hemiplegic gait data with a unified learning rate; and Model D was the final proposed model, which further applied the differential learning rate fine-tuning strategy based on Model C. The ablation results are summarized in Table 4.

**TABLE 4:** Ablation study results of the core mechanisms in the ST-GCN model.

Force component	Model	Pearson’s r	rRMSE (%)
Vertical $\left(F_{z}\right)$	Model A	0.885	11.15
	Model B	0.925	8.95
	Model C	0.965	6.45
	Model D	**0.984**	**5.24**
Anterior–Posterior $\left(F_{y}\right)$	Model A	0.812	15.72
	Model B	0.865	12.84
	Model C	0.925	9.58
	Model D	**0.956**	**8.15**
Medio–Lateral $\left(F_{x}\right)$	Model A	0.745	18.64
	Model B	0.815	15.92
	Model C	0.882	12.45
	Model D	**0.912**	**11.05**

Bold values indicate the best-performing model for each force component and metric.

Compared with Model A, which used only three-dimensional position coordinates and was trained from scratch, Model B achieved higher correlations and lower rRMSE values in all three directions after incorporating velocity, acceleration, and laterality encoding. For example, the rRMSE values for $F_{y}$ and $F_{x}$ decreased from 15.72% to 18.64%–12.84% and 15.92%, respectively. These results indicate that explicit kinematic derivatives and laterality information help the model capture dynamic changes and bilateral differences in hemiplegic gait.

After pretraining on healthy participant data was added, Model C further improved prediction performance, reducing the rRMSE values in the three directions to 6.45%, 9.58%, and 12.45%, respectively. This suggests that the fundamental kinematic–kinetic mapping learned from healthy gait data can provide useful initial representations for GRF prediction in hemiplegic gait. With the additional differential learning rate fine-tuning strategy, Model D achieved the best performance in all directions, with rRMSE values of 5.24%, 8.15%, and 11.05% for $F_{z}$ , $F_{y}$ , and $F_{x}$ , respectively. Overall, the 10-dimensional node features, healthy gait pretraining, and differential learning rate fine-tuning each contributed to performance improvement, with the complete model achieving the best performance for continuous three-dimensional GRF prediction.

## 4 Discussion

This study proposed a continuous three-dimensional ground reaction force (GRF) estimation framework that integrates a spatio-temporal graph convolutional network (ST-GCN), 10-dimensional node-level kinematic features, and two-stage transfer learning for stance-phase kinetic prediction in hemiplegic gait. The results showed that the proposed model predicted vertical, anterior–posterior, and medio–lateral GRF with high waveform agreement under leave-one-subject-out cross-validation and outperformed baseline models, including MLP, 2D-CNN, BiLSTM, and a lightweight Transformer. Further comparisons between the affected and unaffected sides, agreement analysis of clinical discrete features, and ablation experiments indicated that human body topology modeling, explicit kinematic derivatives, laterality encoding, and transfer learning all contributed to improving the model’s ability to represent kinetic characteristics of hemiplegic gait.

### 4.1 Role of human body topology modeling in continuous GRF prediction

Compared with conventional deep learning models that directly flatten marker coordinates or convert them into regular grid-like representations, ST-GCN preserves the spatial connections among human body markers in a graph structure and extracts dynamic changes during the stance phase along the temporal dimension. The results of this study showed that ST-GCN achieved high correlations and low rRMSE values in three-dimensional GRF prediction, particularly for the vertical and anterior–posterior components. This suggests that topology-based spatial feature aggregation helps the model learn the relationship between multi-segment motion and foot–ground interaction forces.

The comparison between the affected and unaffected sides further showed that ST-GCN maintained good prediction performance during stance on both sides. Although the affected side showed relatively higher prediction errors because of the complexity of pathological gait, the model still captured the main waveform characteristics of affected-side GRF. In particular, high Pearson’s r values were retained for affected-side vertical weight-bearing and anterior–posterior propulsion prediction, indicating that the model could identify key kinetic features of hemiplegic gait, such as insufficient weight-bearing and reduced propulsion. These findings suggest that ST-GCN can not only fit overall GRF waveforms but also adapt to side-to-side differences in kinetic patterns in hemiplegic gait.

Nevertheless, the graph architecture may also explain part of the residual prediction error. The adjacency matrix was initialized according to anatomical marker relationships and was shared across participants, which may not fully represent patient-specific compensatory strategies, abnormal inter-segment coordination, or changes in foot–ground contact during hemiplegic gait. Moreover, marker trajectories provide indirect information about external forces and do not directly measure plantar pressure distribution, center-of-pressure progression, or local shoe–floor interaction. These limitations may be particularly relevant for the anterior–posterior and medio–lateral GRF components, which are more sensitive to propulsion, braking, balance control, and abnormal foot placement after stroke.

### 4.2 Contribution of explicit kinematic features and laterality encoding

The ablation study showed that the model trained from scratch using only three-dimensional position coordinates had the lowest performance, whereas adding velocity, acceleration, and laterality encoding improved prediction performance in all three directions. This finding indicates that although position trajectories reflect the spatial changes of markers, velocity and acceleration provide more direct dynamic information for continuous GRF prediction and help the model capture temporal characteristics of kinetic changes during the stance phase.

The introduction of laterality encoding (p) further enhanced the model’s ability to represent asymmetry in hemiplegic gait. The affected and unaffected sides of patients with hemiplegia are not simple mirror images; instead, they differ substantially in weight-bearing, propulsion, and compensatory patterns. By explicitly distinguishing affected-side, unaffected-side, and midline nodes within the node features, the model can more clearly identify the roles of different body regions in kinetic generation, thereby improving its ability to predict asymmetric bilateral GRF characteristics.

### 4.3 Significance of two-stage transfer learning for small hemiplegic gait datasets

The acquisition of hemiplegic gait data is challenging, and sample sizes are often limited. Training deep models directly on small pathological datasets may restrict model generalization. In this study, a two-stage strategy combining pretraining on healthy participants and fine-tuning on patients with hemiplegia was adopted. This allowed the model to first learn the fundamental kinematic–kinetic mapping in typical walking and then adapt to pathological gait characteristics using patient data.

The ablation results showed that adding pretraining on healthy participant data to the 10-dimensional node feature model further reduced prediction errors in all three directions, suggesting that healthy gait data can provide useful initial representations for hemiplegic GRF prediction. With the additional differential learning rate fine-tuning strategy, the complete model achieved the best performance. These results indicate that using a lower learning rate for the spatio-temporal feature extraction backbone and a higher learning rate for the regression head helps preserve general spatio-temporal representations while improving adaptation to hemiplegia-specific kinetic patterns.

Despite these improvements, the remaining errors should be interpreted in light of the domain shift between the healthy pretraining dataset and the hemiplegic target dataset. The two groups differed in age, walking speed, neuromotor control, and gait symmetry, and the data were collected in independent laboratories. Although fine-tuning adapted the pretrained representation to pathological gait, source-domain features learned from healthy walking may not fully capture abnormal foot–ground contact, reduced propulsion, compensatory trunk and pelvic motion, or the large inter-individual variability observed after stroke. In addition, the target dataset included only 30 patients with hemiplegia, which limited the ability of the model to learn the full range of pathological gait phenotypes. Thus, transfer learning improved data efficiency and overall performance, but source–target mismatch and limited target-domain sample size may have contributed to residual prediction errors.

### 4.4 Clinical significance of discrete feature and asymmetry quantification

For hemiplegic gait assessment, the value of a model lies not only in fitting continuous GRF waveforms but also in reliably extracting clinically meaningful discrete kinetic indicators. In this study, the first vertical peak force, anterior–posterior peak propulsive force, and their corresponding asymmetry indices were extracted from the predicted GRF and compared with force-plate measurements using Bland–Altman agreement analysis. The results showed small mean biases for $F_{z1}$ , $F_{y,\max}$ , $ASI_{\mathrm{Fz1}}$ , and $ASI_{Fy,\max}$ , and all participants fell within the 95% limits of agreement.

These findings indicate that ST-GCN can estimate unilateral kinetic peaks and preserve weight-bearing and propulsion differences between the affected and unaffected sides. Because weight-bearing asymmetry and insufficient propulsion are key issues in hemiplegic gait rehabilitation assessment, extracting these discrete metrics from predicted GRF may extend the utility of the model in force-plate-independent, marker-based laboratory gait analysis. Together, the continuous waveform prediction and clinical feature agreement results support the potential use of this method as a tool for quantitative kinetic assessment of hemiplegic gait.

The present results should also be situated carefully relative to existing GRF estimation studies. Some wearable-sensor, load-cell, IMU-based, or multimodal approaches have reported lower errors for selected GRF components or propulsion-related metrics, including in post-stroke walking (Kim et al., 2023; 2025; Revi et al., 2020; Swaminathan et al., 2025). However, direct numerical comparison is difficult because previous studies differ in sensing modality, target force component, walking task, participant population, normalization strategy, and validation design. Therefore, the present findings should not be interpreted as demonstrating universally lower error than existing GRF estimation methods. Rather, the main contribution of this study is the evaluation of a marker-level ST-GCN framework with topology-aware feature extraction and transfer learning for continuous three-dimensional GRF estimation in hemiplegic gait.

The interpretation of these discrete-feature errors should be contextualized against published MDC or SRD values for post-stroke gait. Kesar et al. reported MDC values of 2.85% body weight for peak anterior GRF and 4.65% body weight for mean vertical GRF during treadmill walking in individuals post-stroke (Kesar et al., 2011). Campanini and Merlo further reported that, in stroke patients, the SRD ranged from 5% to 10% of the sample grand mean for vertical GRF-based indices and from 20% to 40% for fore–aft GRF-based indices (Campanini and Merlo, 2009). Because the definitions of these published MDC/SRD metrics do not exactly match all discrete variables extracted in the present study, including $F_{z1}$ , $F_{y,\max}$ , and the ASI metrics, these comparisons should be interpreted cautiously rather than as direct one-to-one equivalence. More recent wearable AP-GRF estimation work in hemiparetic walking has also interpreted estimation errors relative to MDC-based criteria, highlighting the importance of evaluating prediction accuracy against clinically meaningful measurement thresholds (Revi et al., 2020).

In the present study, the 95% limits of agreement for $F_{z1}$ ranged from −5.74% to +3.34% BW, and those for $F_{y,\max}$ ranged from −1.08% to +2.68% BW. These ranges indicate that prediction uncertainty may be comparable to, or exceed, published MDC/SRD thresholds for some GRF-derived metrics. Therefore, although the model preserved overall waveform morphology and group-level bilateral kinetic asymmetry, the current level of error should not be interpreted as sufficient for detecting small within-patient longitudinal changes or for replacing force-plate measurements in individual clinical decision-making. Future work should explicitly optimize and validate the model against MDC- or minimal clinically important difference-based targets for the specific GRF metrics used in hemiplegic gait assessment.

### 4.5 Limitations and future work

This study has several limitations. First, the sample size of patients with hemiplegia was limited, and the model’s generalization across different recovery stages, walking abilities, and clinical phenotypes requires further validation. Future studies should include larger, multicenter datasets and incorporate additional clinical information, such as disease duration, motor function scores, spasticity severity, and assistive device use, to evaluate the applicability of the model across different hemiplegic subtypes.

Second, although the proposed framework does not require force-plate input during inference, it is not laboratory-free because the present model still requires marker-based optical motion capture for feature extraction. The use of 39 Plug-in Gait markers provided high-resolution whole-body kinematic information and enabled the model to learn spatial coordination and pathological asymmetry during hemiplegic gait. However, optical motion capture systems are expensive, require specialized space and technical expertise, and are not available in most routine clinical or community rehabilitation settings. Therefore, the current implementation should be interpreted primarily as a force-plate-independent, marker-based laboratory framework or as an intermediate step toward more accessible kinetic gait assessment, rather than as a directly deployable clinical monitoring tool.

A feasible pathway toward clinical deployment would require replacing or complementing the marker-based input with more accessible sensing modalities. Future studies may adapt the proposed graph-based framework to markerless visual motion capture, reduced marker sets, IMU-based kinematics, pressure-sensing insoles, or multimodal wearable systems. In this process, model robustness must be re-evaluated under realistic sources of error, including marker or key-point occlusion, soft-tissue artifacts, sensor drift, sensor placement variability, missing data, and slower or more asymmetric walking patterns in patients with stroke. Additional multicenter validation and real-time testing will also be necessary before the method can be considered for clinical decision support or home-based rehabilitation monitoring. In addition to sensing accuracy, future deployment of wearable or home-based gait assessment systems will require secure health-data management, privacy protection, and user engagement mechanisms. Recent studies have suggested that blockchain with zero-knowledge proofs may improve privacy preservation and data integrity in wearable health technologies (Khan et al., 2025a), while generative AI and gamification may support real-time biosensor monitoring and patient engagement in digital health applications (Khan et al., 2025b).

Finally, this study mainly performed offline validation during level walking at self-selected speed and did not cover complex tasks such as stair negotiation, slope walking, or turning. Future work may extend the framework to multi-scenario gait data and evaluate its potential for long-term rehabilitation monitoring and real-time kinetic assessment.

## 5 Conclusion

This study addressed the problem of estimating continuous stance-phase three-dimensional GRF in post-stroke hemiplegic gait from marker-level kinematic data without using force-plate signals as model input. Within an offline laboratory setting, the proposed force-plate-independent, marker-based framework integrated an ST-GCN, 10-dimensional node-level kinematic features, pathological laterality encoding, and two-stage transfer learning.

LOSO-CV based on 30 patients with hemiplegia showed that the proposed model reconstructed overall GRF waveform morphology with high agreement for the vertical, anterior–posterior, and medio–lateral components and outperformed the baseline models evaluated in this study. The predicted GRF also preserved clinically relevant group-level kinetic features, including vertical loading, anterior–posterior propulsion, and bilateral asymmetry.

These findings support the value of topology-aware marker modeling, explicit kinematic derivatives, laterality encoding, and healthy-to-hemiplegic transfer learning for kinetic estimation in hemiplegic gait. However, the current framework should be interpreted as a laboratory-based kinetic assessment approach rather than a fully wearable or home-based clinical monitoring system. Future studies should validate the method in larger multicenter cohorts and further examine markerless, wearable, or reduced-marker implementations for real-world rehabilitation applications.

## Data Availability

The raw data supporting the conclusions of this article will be made available by the authors, without undue reservation.
